# A Study of Molecular Signals Deregulating Mismatch Repair Genes in Prostate Cancer Compared to Benign Prostatic Hyperplasia

**DOI:** 10.1371/journal.pone.0125560

**Published:** 2015-05-04

**Authors:** Sanmitra Basu, Subhadipa Majumder, Ankur Bhowal, Alip Ghosh, Sukla Naskar, Sumit Nandy, Subhabrata Mukherjee, Rajan Kumar Sinha, Keya Basu, Dilip Karmakar, Soma Banerjee, Sanghamitra Sengupta

**Affiliations:** 1 Department of Biochemistry, University of Calcutta, Kolkata, West Bengal, India; 2 Department of Pathology, Calcutta National Medical College & Hospital, Kolkata, West Bengal, India; 3 Department of Urology, Calcutta National Medical College & Hospital, Kolkata, West Bengal, India; 4 Centre for Liver Research, Institute of Post-Graduate Medical Education & Research, Kolkata, West Bengal, India; Queen Mary Hospital, HONG KONG

## Abstract

Prostate cancer is one of the leading causes of mortality among aging males. There is an unmet requirement of clinically useful biomarkers for early detection of prostate cancer to reduce the liabilities of overtreatment and accompanying morbidity. The present population-based study investigates the factors disrupting expression of multiple functionally related genes of DNA mismatch repair pathway in prostate cancer patients to identify molecular attributes distinguishing adenocarcinoma from benign hyperplasia of prostate. Gene expression was compared between tissue samples from prostate cancer and benign prostatic hyperplasia using real-time-PCR, western blot and immunohistochemistry. Assessment of genotypes of seven single-nucleotide-polymorphisms of three MMR genes was conducted using PCR-coupled RFLP and sequencing. Promoter methylation was interrogated by methylation-specific-PCR and bisulfite-sequencing. Interaction between microRNAs and MMR genes was verified by 3'UTR-based dual luciferase assays. Concurrent reduction of three MMR genes namely *hMLH1*, *hMSH6* and *hMSH2* (34-85%, P<0.05) was observed in prostate cancer tissues. *hMSH6* polymorphism rs1800932(Pro92Pro) conferred a borderline protection in cancer patients (OR = 0.33, 95% CI = 0.15-0.75). Relative transcript level of *hMLH1* was inversely related (r = -0.59, P<0.05) with methylation quotient of its promoter which showed a significantly higher methylation density (P = 0.008, Z = -2.649) in cancer patients. hsa-miR-155, hsa-miR-141 and hsa-miR-21 gene expressions were significantly elevated (66-85%, P<0.05) in tumor specimens and negatively correlated (r = -0.602 to -0.527, P<0.05) with that of MMR genes. hsa-miR-155 & hsa-miR-141 and hsa-miR-155 & hsa-miR-21 were demonstrated to bind to their putative seed sequences in *hMLH1* and *hMSH6* 3’UTRs respectively. Relatively higher expression of DNA methyl-transferases (*DNMT1* and *DNMT3b*) and *HIF-1α* genes (34-50%, P<0.05) were also detected in tumor tissues. This study provides statistical evidence that MMR deficiency is correlated with hypermethylation of *hMLH1* promoter and upregulation of hsa-miR-155, hsa-miR-141 and hsa-miR-21 in prostate cancer. This comparative study reflects that microRNA expression level, particularly hsa-miR-155, exhibits predictive signature of prostate adenocarcinoma.

## Introduction

Prostate cancer is a complex multi-factorial disease characterized by an array of clinical phenotypes ranging from slow growing indolent tumors to aggressively metastatic lesions. Rate of prostate cancer incidence varies by over 25-fold globally with Australia, New Zealand, Europe and Northern America having the highest rates [1,2]. The age-adjusted incidence rates in the Asian countries are low and range between 2.3 to 9.0 per 1,00,000 [3–6]. Overall, the rate of prostate cancer incidence in India is low (3.7/1,00,000) [7,8]. In recent past, with the increased migration of rural population to urban areas, changing life styles, increase in the proportion of elderly persons in the population and improvement in awareness and disease surveillance, there has been a rise in the absolute number of new prostate cancer patients in the age group above 65 years in the metropolitan cities of India [8–10]. According to the most recent Population Based Cancer Registries (PBCRs) of different cities for the time period 2008–2011, prostate is the second leading site of cancer among males in large Indian cities like Delhi, Kolkata, Pune and Thiruvananthapuram and third leading site of cancer in cities like Bangalore and Mumbai [11]. Since an increase in incidence rate is correlated with increased mortality rate particularly in countries with lower resource setting, the current situation underscores the necessity for an accurate and early detection of the disease.

Genome-wide and candidate gene based association studies in prostate cancer have attempted to elucidate the role of common risk alleles affecting disease susceptibility and aggressiveness [12–14]. But many of these were inconclusive as a sizable fraction of disease-associated SNPs were located in non-coding region while some of them suffered from lack of replicability in other populations [15–17]. Non-reproducibility of genetic association studies may be explained by multiple confounding factors such as, (a) a difference in risk allele frequency between populations, (b) divergent association of an allele with risk in different populations, and (c) possible interaction of an allele with other genetic or environmental factors that vary among populations [18]. This highlights the importance of an approach that explores the biological association of cancer risk alleles which may essentially be broadly consistent across ethnic groups.

Benign prostatic hyperplasia (BPH) is another common clinical syndrome in aging men which often coexists with prostate cancer. Prostate cancer and BPH have striking similarities with respect to hormone-dependent growth, response to anti-androgen therapy and risk factors such as prostate inflammation and metabolic disruption [19]. More than 80% of men with prostate carcinoma also have a BPH component although the latter is not considered to be a precursor [20–22]. A Swedish nationwide population-based record-linkage data analysis among 86,626 men showed that selection of BPH treatment modalities also has significant impact on prostate cancer risk and mortality [22]. Over the past few decades the serum prostate specific antigen (PSA) level screening has been used as a tool for initial surveillance of prostatic diseases. Since PSA is not a reliable screening tool, urologists, nowadays, use different pre-treatment risk stratification models, which combine serum PSA level, clinical staging and biopsy Gleason score in order to define pathological stages and predict the risk of disease recurrence following definitive local treatment [23–26]. Unfortunately, these stratification models do not take into account the genetic and molecular characteristics featuring the tumors in the prostate gland. The high incidence of prostate cancer in many parts of the world and its frequent co-existence with BPH demand for identification of molecular criteria that may be incorporated in the risk prediction tools of prostate cancer to reduce overtreatment and economic burden on the healthcare system in many countries including India.

DNA mismatch repair (MMR) system plays a ubiquitous role in ensuring replication fidelity that is central to preservation of genomic integrity [27]. Defects in MMR genes confer a mutator phenotype with small genetic disruptions leading to emergence of somatic mutations that may be significant for initiation of late onset diseases like prostate cancer [28]. Approximately 73% of prostate tumors are carriers of MMR gene mutations, which could be equated to carriers having a 3.67-fold (95% CI, 2.32–6.67) increased risk of prostate cancer [29]. A number of population-based case-control studies have indicated the possible association of different MMR polymorphisms with susceptibility, aggressiveness and recurrence of prostate cancer [30–33]. However, it is still controversial whether men with Lynch syndrome are at heightened risk of prostate cancer. To address these conundrums, the present study compares MMR gene expression in BPH and prostate cancer patients and appraises the factors regulating the pattern. Apart from the heritable or somatic mutation, non-genetic chemical aberration such as abnormal promoter methylation and dysregulation of microRNAs are increasingly recognized as part of aging and age-related pathogenesis [34–36]. Therefore, to explore the regulatory factors underlying MMR gene expression, in addition to genetic epidemiology, we investigated methylation status of MMR gene promoters. Since, altered microenvironment in the aging prostate gland may affect the repertoire of noncoding regulatory RNAs, we also studied the expression of oncomiRs namely hsa-miR-21, hsa-miR-141 and hsa-miR-155 and their interaction with the 3’UTR regions of MMR genes under study. The findings presented in this comparative evaluation mirrored the heterogeneous events occurring in patients’ tissue specimens and can be utilized to develop a cost-effective biomarker if incorporated as a tool in cancer prediction model.

## Materials and Methods

### Recruitment of patients

Age and ethnicity matched male patients with prostate cancer (n = 104) and BPH (n = 186) were recruited from Departments of Urology from Saroj Gupta Cancer & Research Institute, Thakurpukur, Kolkata and Calcutta National Medical College and Hospital, Kolkata in the periods between November 2007 to September 2012 and December 2012 to October 2013 respectively. All patients were new cases with no medical history of chemotherapy or surgery. The patients were then examined by a panel of expert urologists and evaluated according to standard imaging procedures and laboratory analyses for prostate cancer. Information on demographic and family histories and clinical parameters such as serum PSA, blood sugar, past infections and biopsy report confirming the malignancy and Gleason score had been recorded from all study participants. Patients with prostatitis and high-grade prostatic intraepithelial neoplasia were excluded. Two ml of venous blood had been collected from all study participants and used for DNA isolation. Tissue samples were collected from a subset of BPH patients (n = 25) undergoing trans-rectal ultrasound (TRUS) guided biopsy or transurethral resection of prostate (TURP). On the other hand, tissue samples were collected from a subset of 25 prostate cancer patients who underwent TRUS-guided biopsy. Prior to sample processing for further molecular experiments, a pair of needle biopsy specimen was collected from the same region of a patient, one of which was subjected for pathological justification of tissue composition to detect any cancerous lesions. Three pathologists independently examined the histological features of each tissue specimen after hematoxylin and eosin staining. Cancer samples with 70% tumor cell content and Gleason score between 6 and10 were included in this study. The other section was stored at -80°C for future molecular biological analyses. Relevant clinical and demographic details of all the study participants including those where from tissue samples were collected have been summarized in Table 1.

**Table 1:** Clinical and demographic characteristics of the study participants

Characteristics	BPH	Prostate Cancer	P value	Logistic regression analyses Estimate (P values)
**Genetic Epidemiology**				
**Number of subjects**	186	104		
**Mean Age(years)**	67.33+7.7	68.5+8.88	0.241	3.952e-18(0.691)
**Serum PSA level**	6.04+10.89	73.57+84.00	0.0001*	6.478e-17(0.541)
**Prostate volume**	52.57+25.37	83.13+32.36	0.001*	-9.093e-20(0.958)
**Metastasis**		n = 16		
**Gleason Score**		< 6: n = 29 (27.9%); 7: n = 24 (23.07%); >8: n = 51 (49.03%)		
**Gene expression and promoter methylation**				
**Number of subjects**	25	25		
**Mean Age(years)**	69 +7.13	71.71+9.63	0.263	
**Serum PSA level**	5.8+6.5	73.4+38.57	0.0001*	
**Prostate volume**	92.97+72.89	73.11+30.69	0.215	
**Metastasis**		n = 04		
**Gleason Score**		< 6: n = 07 (28.0%); 7: n = 08 (32.0%); >8: n = 10 (40.0%)		

*P value significant at 0.05.

### Ethics statement

Each patient contributed to the study signed a written informed consent. The study was approved by the University Biosafety and ethics committee, University of Calcutta and had been carried out following the ethical principles for medical research involving human subjects mentioned in declaration of Helsinki developed by World Medical Association (WMA).

### Gene Expression assay

Total RNA (1μg) extracted from tissue (>50 mg) using TRI reagent (Sigma Aldrich) was reverse transcribed using random hexamers and High-Capacity cDNA Reverse Transcription Kit (Applied Biosystems Inc.). The optimized condition for cDNA preparation was 10 mins at 25°C, 120 mins at 37°C followed by heating at 85°C for 5 mins in a thermal cycler (Applied Biosystems GeneAmp PCR System 9700) and stored at -20°C. 18S rRNA and *β-actin (ACTB)* were used as endogenous controls. A 1:10 fold dilution of cDNA samples were used as the template and all quantitative PCR reactions were carried out in a 10 ml reaction volume with 5 ml of (2X) Maxima SYBR Green/ROX qPCR Master Mix (Thermo Scientific) with optimized concentrations of specific primers using Applied Biosystems 7900HT Fast Real-Time PCR System (S1 Table). The thermal cycler was programmed for an initial denaturation step of 5 min at 95°C followed by 40 thermal cycles of 30 sec at 95°C, 30 sec at 60°C and 30 sec at 72°C.The experiments were carried out in triplicate to ensure best reproducibility including the non-template controls each time following MIQE criteria. Specificity of PCR amplification for each primer pair was confirmed by melting curve analysis.

### Western blot

Protein extracts (30–40 μg/lane) prepared from 100 mg of frozen tissue from 12 randomly selected samples (n = 6 for each group) from the gene expression panel using RIPA buffer and Protease inhibitor cocktail (Sigma) were resolved in SDS-PAGE using PageRuler Plus Prestained Protein Ladder (Thermo Scientific) and transferred to polyvinylidene fluoride membrane (Pall Corporation). The primary antibodies used included rabbit polyclonal anti-MLH1 (C-20: sc-582), anti-MSH2 (N-20: sc-494), goat polyclonal anti-MSH6 (N-20: sc-1243), mouse monoclonal anti-HIF-1α (28b: sc-13515) and anti-β-actin (anti-ACTB) (C4: sc-47778) (Santa Cruz Biotechnology). Band intensity estimation and quantification were done using ImageJ software (http://imagej.nih.gov/ij/). Average optical density of the MMR proteins for a sample was normalized with respect to that of ACTB.

### Immunohistochemistry

Paraffin-embedded tissue sections of size 5 μm were deparaffinized and rehydrated. Following antigen-retrieval, nonspecific binding and endogenous peroxidase were blocked by incubating sections in PBS with BSA and a solution of H_2_O_2_ in methanol successively. Tissue sections (n = 4 for each group) were incubated with primary antibody overnight at 4°C in a moist chamber, washed and incubated with corresponding secondary antibody. Peroxidase activity was visualized with 3,3'-diaminobenzidine. After hematoxylin counterstaining, sections were dehydrated and mounted in Distyrene Plasticizer Xylene. In negative controls PBS substituted primary antibody. The slides were examined under light microscope with 400X magnification. The primary antibodies used were the same as those used for Western blot.

### Genetic epidemiology

Approximately 100 ng of genomic DNA isolated from Buffy coat of blood samples (2 ml) using DNeasy blood and tissue kit (Qiagen) was amplified with primer pairs specific for seven single nucleotide polymorphisms (SNPs) (S2 Table). Genotypes were assigned by digesting the PCR products with 10U of respective restriction endonucleases (NEB Inc.) as per manufacturer’s instructions followed by agarose gel (1.5–2%) electrophoresis. To confirm the genotypes ascribed by RFLP, PCR products from 15% of the total sample were subjected to sequencing using Big-Dye Terminator v3.1 and ABI Prism-3100 Genetic Analyzer (Applied Biosystems). Locus rs1799977 was genotyped directly by sequencing.

### Promoter methylation assay

CpG islands were mapped using CpGplot (http://www.ebi.ac.uk/Tools/seqstats/emboss_cpgplot/) along 1 kb upstream of the transcriptional start site (TSS) of all three MMR genes. DNA (1μg) isolated from tissue samples using DNeasy blood and tissue kit were subjected to bisulfite treatment with Epitect Bisulfite kit (Qiagen). A specific nested-PCR was performed using products from first amplification reaction and methylation specific primers with bisulfite-modified DNA from tissue samples (S1 Table). Negative control samples with water were included for each set of PCR reaction to check for PCR contamination. To establish methylation-specific PCR (MSP), the technique was standardized by using different concentrations of bisulfite modified DNA (e.g. 50, 100, 200 and 250 ng) as starting material to identify a threshold minimum concentration that produced positive result. Once the DNA concentration was optimized, MSP was repeated for different number of cycles (e.g. 30, 35, 38 and 40 cycles) to standardize the PCR protocol. DNA from normal leukocytes treated in vitro with SssI methyltransferase (NEB Inc.) following manufacturer’s protocol was used as a positive control for the methylated alleles. DNA from normal leukocytes that was not bisulfite-modified was used as negative control for methylation. Each PCR product (10μl) was directly loaded onto 2% agarose gel, stained with ethidium bromide and visualized directly under UV illumination. Quantitative assessment of methylation status of *hMLH1* promoter was performed with bisulfite-modified DNA in 7900 HT instrument, using SYBR Green/ROX qPCR Master Mix (Thermo Scientific). A region devoid of CpG islands was amplified from *ACTB* locus that served as endogenous control in qMSP. Leukocytes DNA (5 μg) from a healthy individual was fully methylated in vitro with 20 U SssI methyltransferase (NEB Inc.) and its serial dilutions (90–0.009 ng) were used to construct a calibration curve for each plate. Negative controls without template and positive controls with completely methylated DNA were included in each set of PCR assays. The relative level of *hMLH1* promoter methylation for each sample was normalized with respect to that of *ACTB* [(*MLH1/ACTB*) x 1000] and log-transformed value of this measure was referred to as methylation quotient (MQ). Bisulfite-modified DNA (100 ng) isolated from tissue (n = 50) and Buffy coat (n = 30) was amplified using methylation-specific primers followed by sequencing of the PCR products. Bisulfite sequencing of *hMLH1* promoter for leukocyte samples was carried out for the same set of samples employed in qMSP experiments and the cancer patients included in this assay had localized tumor.

### 3'UTR assay

3'UTR sequences from *hMLH1* (326bp), *hMSH2* (485bp) and *hMSH6* (352bp) genes encompassing target sites for microRNAs under study were amplified using specific primers containing recognition sites for XhoI and NotI, cloned into pTZ57R/T vector (InsTAclone PCR Cloning Kit, Fermentas) and subcloned into pSiCHECK2 (S1 Table). Approximately 100 bp upstream and downstream sequences flanking the 70 nucleotide pre-miR sequences (http://www.genecards.org/) were amplified for hsa-miR-21, hsa-miR-141 and hsa-miR-155 using appropriate primers with BamHI and HindIII recognition sites and cloned into pRNAU6.1 vector (S1 Table). PC3 and HepG2 cell lines obtained from National Centre for Cell Science, Pune, India were maintained in RPMI 1640 and DMEM media respectively containing 10% (v/v) fetal calf serum (Gibco BRL), 100 units/ml penicillin, 100 mg/ml streptomycin in a humidified 5% CO_2_ chamber. Cells (10^5^) were seeded 14–16 hrs before transfection, transiently transfected with empty pSiCHECK2 and cotransfected with 0.1 mg/ml of the 3'UTR construct in pSiCHECK2 with either 0.25 mg/ml of empty pRNAU6.1 or with pre-microRNA construct in pRNAU6.1. Cells were lysed after 48 hrs and Firefly and Renilla luciferase activities were evaluated using DLR assay system in a GloMax 20/20 Luminometer (Promega). Renilla luciferase activity was normalized with respect to Firefly luciferase activity and total protein produced was estimated by Bradford method. All transfection assays were done in triplicate. The change in normalized luciferase expression was denoted as percentage RLU relative to the control.

### MicroRNA expression

To find out the potential microRNA binding sites, 3'UTR region of a gene was scanned using TargetScan (www.targetscan.org/), miRBase (www.mirbase.org/), microRNA.org (www.microrna.org/), RegRNA (www.regrna.mbc.nctu.edu.tw/) and MicroCosm (www.ebi.ac.uk/enright-srv/microcosm/). Target microRNAs were selected based on its conserved seed match or seed match with a higher context score. Total RNA (1μg) isolated from tissue samples were reverse transcribed using miScript PCR starter kit (Qiagen) according to manufacturer’s protocol and microRNAs were quantified using miScript SYBR Green PCR kit and 10X miScript Primer Assay (specific for microRNA of interest, Qiagen). U6 small nuclear 2 was quantified to normalize the levels of microRNA expression using Hs_RNU6B_2 miScript Primer Assay (Qiagen).

### Statistical Analysis

Expression of MMR and microRNA genes was quantified in terms of RQ (Relative Quantification = 2^-ΔΔCq^) value where ∆C_q_ expression of a cancer sample is normalized to BPH control. Percentage reduction in mRNA level calculated as (1-∆∆C_q_)*100 was represented by bar diagram and analyzed by Student-t test in GraphPad Prism (http://www.graphpad.com). Pairwise Pearson’s correlation co-efficients and corresponding P values (two-tailed) were calculated using the ΔC_q_ values representing MMR and microRNA transcripts under study. Genotype and allele frequencies of polymorphic loci were computed by gene counting. Any departure from Hardy–Weinberg equilibrium (HWE) for a locus was examined using HaploView (http://www.broadinstitute.org/scientific-community/science/programs/medical-and-population-genetics/haploview/haploview). Genetic association was tested using logistic regression with adjustment for covariates such as age, serum PSA level and prostate volume. Association of a SNP with prostate cancer risk at genotype levels was evaluated under co-dominant (11 vs 12 vs 22) and dominant (12+22 vs 11) models using 3-way and 2-way contingency tables respectively. Allele frequencies of each locus from BPH and cancer samples were compared using a 2-way contingency table. Statistical significance defined as P value for each comparison was corrected using Benjamini-Hochberg procedure [37]. Studies on linkage disequilibrium (LD) were performed with the genotypic frequencies of the loci under study using HaploView. MicroRNA-mediated repression of MMR gene expression in terms of luciferase activity was tested using Student-t test in GraphPad Prism (http://www.graphpad.com/). A heat map displaying variations in intensity on a color scale representing relative transcript levels of different genes was generated using dChip (http://www.biostat.harvard.edu/~cli/dchip_2010_01.exe).

## Results

### Evaluating MMR gene expression

**Fig 1:**
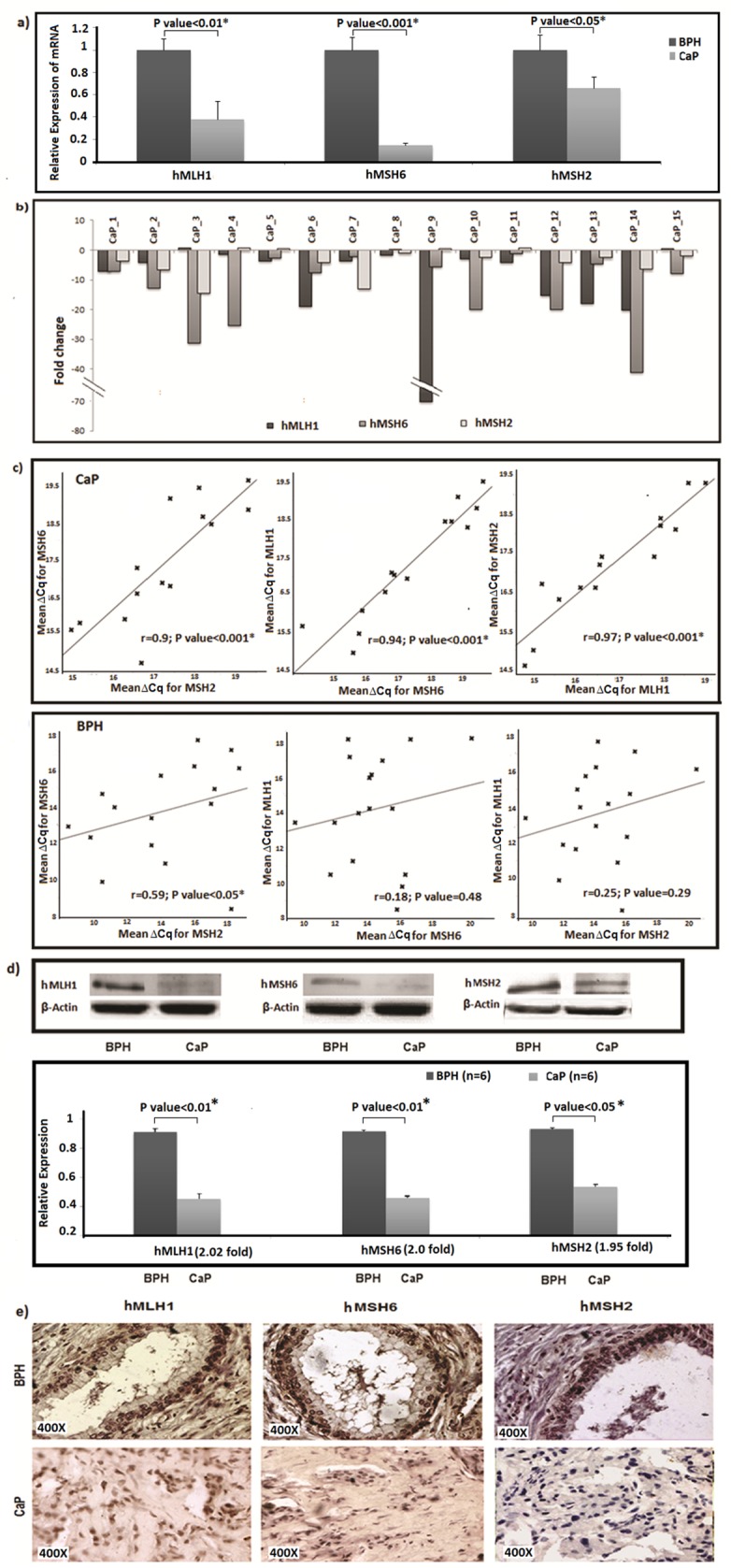
MMR gene expression in cancer and benign hyperplasia of prostate glands. (a) Bar diagram showing decreased expression of transcripts of *hMLH1*, *hMSH6* and *hMSH2* genes in prostate cancer tissues compared to that of BPH patients. 18S rRNA was used as endogenous control. * indicates P <0.05. (b) Fold differences of *hMLH1*, *hMSH6* and *hMSH2* expression in each of 15 cancer tissue specimens with respect to population mean of ∆C_q_ estimate of the said gene in BPH tissues. (c) Upper panel: Plots showing correlation between transcript levels of *hMSH6* and *hMSH2*, *hMSH6* and *hMLH1* and *hMSH2* and *hMLH1* in prostate cancer. Lower panel: Plots showing correlation between transcript levels of *hMSH6* and *hMSH2*, *hMSH6* and *hMLH1* and *hMSH2* and *hMLH1* in BPH. Pearson’s correlation coefficient (r) and P values are indicated for each test. * indicates P <0.05. (d) Upper panel: Representative result of Western blot for MMR proteins in cancer and benign tissue of prostate. β-Actin acted as endogenous control. Lower panel: Bar diagram showing difference in expression of *hMLH1*, *hMSH6* and *hMSH2* at the protein level between BPH and prostate cancer tissue samples. Fold changes and statistical significance are indicated. * indicates P <0.05 and n indicates the number of samples. (e) Photomicrograph of representative prostate archival specimens immune-stained with antibodies against hMLH1, hMSH6 and hMSH2 at 400x magnification showing decreased expression of the MMR genes in prostate cancer tissues compared to benign hyperplastic tissues.

The evaluation of relative transcript levels with respect to 18S rRNA in a sample size of 15 prostate cancer and 15 BPH patients revealed a significantly lower expression of *hMLH1* (62%; P value<0.01), *hMSH6* (85%; P value<0.001) and *hMSH2* (34%; P value<0.05) genes in tumor tissues (Fig 1A). A similar pattern of reduction in MMR transcript levels was observed in tumor samples using *ACTB* as endogenous control (S1 Fig). To detect the proportion of tumor samples showing concomitant down-regulation of MMR genes, the ∆C_q_ estimate of individual MMR transcript of each cancer sample was compared with mean ∆C_q_ estimate of the of 15 BPH patients. The relative expression of *hMLH1*, *hMSH6* and *hMSH2* genes was diminished in 13, 14 and 11 patients respectively (Fig 1B). Reduction of gene expression was significantly correlated in all three pairwise combinations in cancer tissues, namely *hMLH1* and *hMSH2* (r = 0.97, P value<0.001), *hMSH2* and *hMSH6* (r = 0.9, P value<0.001) as well as *hMSH6* and *hMLH1* (r = 0.94, P value<0.001) in cancer tissues (Fig 1C, upper panel). In BPH tissues expressions of only *hMSH6* and *hMSH2* genes were weakly correlated (r = 0.6, P value<0.05) (Fig 1C, lower panel). A semi-quantitative densitometric measurements of band intensities from the Western blot assays confirmed the down-regulation of hMLH1 (2.02 fold, P value<0.01), hMSH6 (2 fold, P value<0.01) and hMSH2 (1.95 fold, P value<0.05) proteins in tumor samples (n = 6) in comparison with BPH samples (n = 6) (Fig 1D). The reduced expression of MMR genes was also validated in immunohistochemical analysis of representative tissue samples collected from prostate cancer and BPH patients. Immunohistograms depicted hMLH1 and hMSH2 expressions predominantly nuclear while hMSH6 staining was observed both in nucleus and cytoplasm. (Fig 1E).

### Dissecting the factors regulating MMR gene expression

#### Genetic association of MMR gene polymorphisms

To address whether dysregulated expression of MMR genes in tumor tissues was attributed to any germline polymorphisms, the genotype and allele frequencies of seven tagSNPs located within 1 kb upstream from the transcriptional start sites and in the coding sequences of *hMLH1*, *hMSH6* and *hMSH2* genes were selected for their possible genetic association with prostate cancer risk. These include rs1800734, rs1799977 of *hMLH1*, rs2303425, rs6753135 of *hMSH2* and rs3136228, rs1042821 and rs1800932 of *hMSH6*. rs6753135 was found to be monomorphic in our study population. Genotype frequencies of remaining loci were in accord with HWE in cancer and BPH samples. To study the effect of confounding factors on prostate cancer risk, logistic regression analyses were performed which resulted in P values of 0.691, 0.541, 0.968 for age, log-transformed serum PSA level and prostate volume respectively. Therefore, for subsequent association analyses, the data was not corrected for the above covariates. The allele and genotype frequency distribution for none of the *hMLH1* and *hMSH2* SNPs varied significantly between cancer and BPH patients in the present cohort. A statistically significant difference in genotypic proportions between two study groups was observed for *hMSH6* Pro92Pro polymorphism under co-dominant (χ2 = 10.5, P value = 0.03) and dominant (χ2 = 9.36, P value = 0.012) models (Table 2). The significance was not retained following Benjamini-Hochberg correction when allelic proportions of the locus were compared between the groups (χ2 = 6.56, P value = 0.06; OR = 0.388 [0.18–0.82]). AG and GG genotypes together was associated with a decreased risk of prostate cancer (χ2 = 9.36, P value = 0.012; OR = 0.33 [0.15–0.75]) compared to the reference AA genotype of rs1800932.

**Table 2:** Genotype and allele based association of MMR gene polymorphisms with prostate cancer risk.

Gene-dbSNP ID position	Control/Case	Genotype Proportion			MAF+STDEV	Comparison of allelic proportions 2 (P value)	Comparison of genotypic proportions 2 (P value)	
		11	12	22			11vs12vs22	(12+22)vs11
MLH1-rs1800734 (-93G>A)	BPH	0.42	0.5	0.08	0.33±0.003	0.53 (0.620)	0.94 (0.620)	0.174 (0.677)
	Cancer	0.38	0.5	0.115	0.36 ±0.005			
MLH1-rs1799977 Ile121Val(A>G)	BPH	0.77	0.2	0.03	0.13 ±0.002	2.1 (0.336)	2.171 (0.506)	0.914 (0.408)
	Cancer	0.83	0.16	0.009	0.09 ±0.003			
MSH2-rs2303425 (-118T>C)	BPH	0.86	0.14	0	0.071 ±0.001	0.24 (0.620)	0.434 (0.612)	0.909 (0.408)
	Cancer	0.88	0.12	0	0.057 ±0.002			
MSH6-rs3136228 (-557G>T)	BPH	0.8	0.18	0.02	0.102±0.002	4.61 (0.270)	4.13 (0.260)	3.65 (0.112)
	Cancer	0.7	0.28	0.02	0.16±0.0035			
MSH6-rs1042821 Gly39Glu(G>A)	BPH	0.59	0.36	0.05	0.218±0.003	2.4 (0.338)	6.56 (0.120)	6.45 (0.033)*
	Cancer	0.73	0.21	0.06	0.16±0.0036			
MSH6- rs1800932 Pro92Pro(A>G)	BPH	0.8	0.2	0	0.104±0.0016	6.56 (0.270)	10.5 (0.030)*	9.36 (0.012)*
	Cancer	0.92	0.07	0.01	0.043±0.0019			

MAF denotes minor allele frequency and STDEV denotes standard deviation.

*P<0.05.

#### CpG hypermethylation of MMR gene promoter

**Fig 2:**
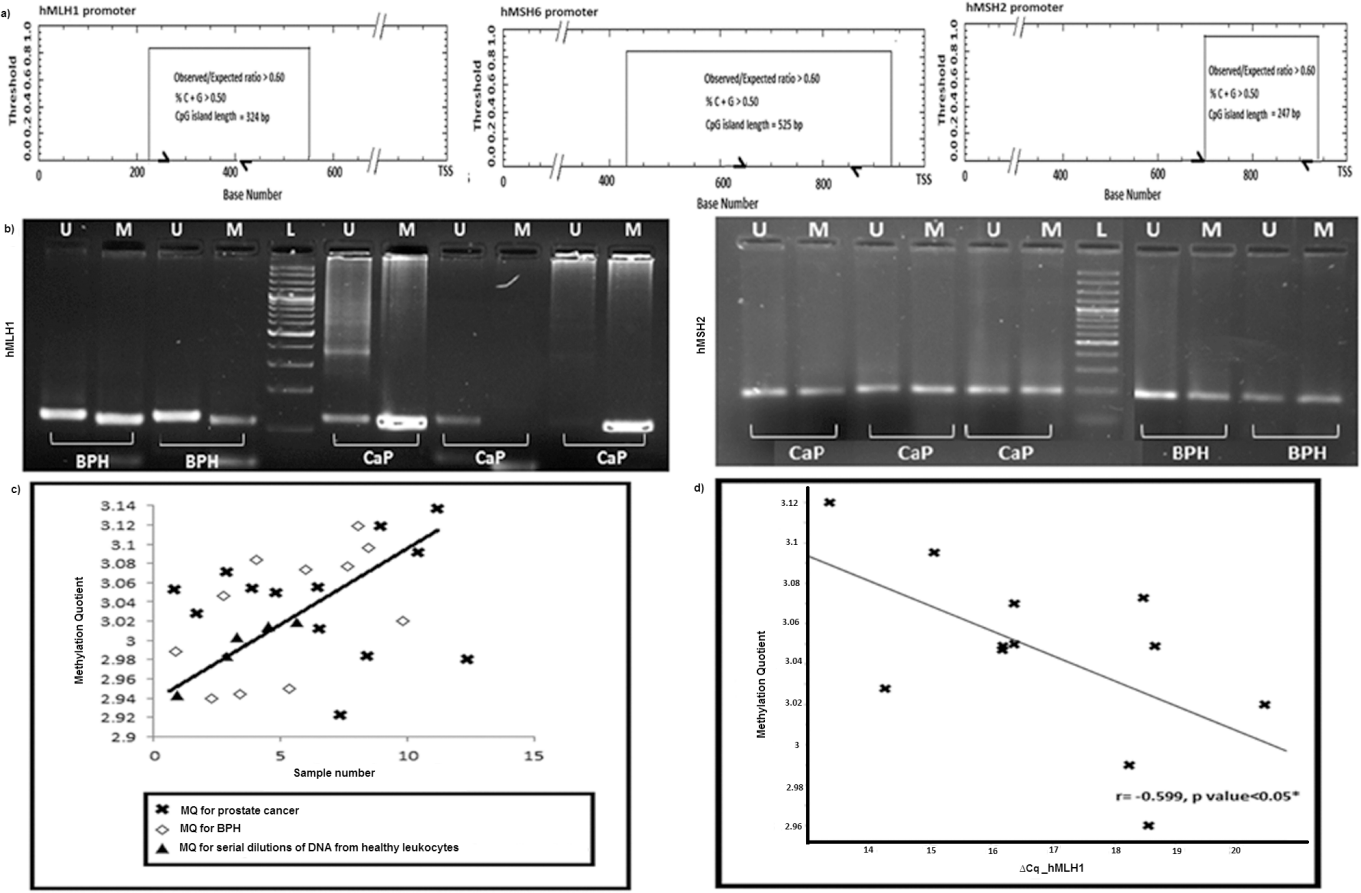
Promoter hypermethylation of MMR genes. (a) Diagram showing the *in silico* mapping of putative CpG islands in the promoter region of *hMLH1*, *hMSH6* and *hMSH2* genes with position of forward and reverse primers demarcated as half arrows which were used in methylation assays. TSS indicates the transcriptional start site.(b) Gel photograph illustrating the methylation status of *hMLH1* and *hMSH2* promoter region CpG islands in prostate cancer and BPH tissues as determined by methylation-specific PCR in the left and right panels respectively. Primer sets for amplification were designated as unmethylated (U) and methylated (M).The presence of PCR product in lanes marked U indicates unmethylated *hMLH1* and *hMSH2*; product in lanes marked M indicates hypermethylated *hMLH1* and *hMSH2*. L indicates 100 bp plus ladder. (c) Distribution of methylation quotients [MQ = (logMLH1/ACTB) X1000] obtained from analyzing prostate cancer and BPH tissue DNAs using qMSP. The calibration curve was generated to determine quantitative accuracy of qMSP with five different dilutions of in vitro fully methylated DNA from normal healthy human lymphocytes.(d) Plot showing correlation between transcript levels of *hMLH1* (∆C_q_) in prostate cancer tissue samples with methylation quotients corresponding *hMLH1* promoter. Pearson’s correlation coefficient (r) and P values are indicated for each test. * indicates P <0.05.

Next, we investigated possible influence of promoter methylation and microRNAs on the observed transcriptional aberration of MMR genes. A map of putative CpG islands in promoter region of the MMR genes was constructed (Fig 2A). The overall C+G content in the regions selected for evaluating methylation status were 68% (324 bp), 65% (247 bp) and 79% (472 bp) respectively. A semi-quantitative estimation of methylation status was carried out using bisulfite-modified genomic DNA isolated from 15 prostate cancer and 15 BPH tissues by MSP using nested primers. This patient pool was the same as that was employed in the mRNA expression assays. Thirteen prostate cancer samples yielded PCR products using methylation-specific primers for *hMLH1*, four of which were also amplified with primers specified for unmethylated sequences. The genomic DNA from all 15 BPH patients yielded PCR products using methylation-specific *hMLH1* primers. Five BPH samples were also amplified with unmethylated primers. All 30 bisulfite-modified DNA samples were amplified using both methylation and unmethylation-specific primers for *hMSH2* promoter (Figs 2B and S2). None of the samples were amplified with methylation-specific primers embedding *hMSH6* promoter. Quantitative analysis of methylation profile was exclusively pursued for *hMLH1* gene promoter which displayed a differential behavior. Although the distribution of MQs and average MQ estimates between prostate cancer (3.00±0.06) and BPH (3.04±0.04) patients did not differ significantly (Fig 2C), the relative transcript level of *hMLH1* was shown to correlate inversely with MQ estimates in prostate cancer patients (r = -0.599; P value <0.05) only (Fig 2D).

**Fig 3:**
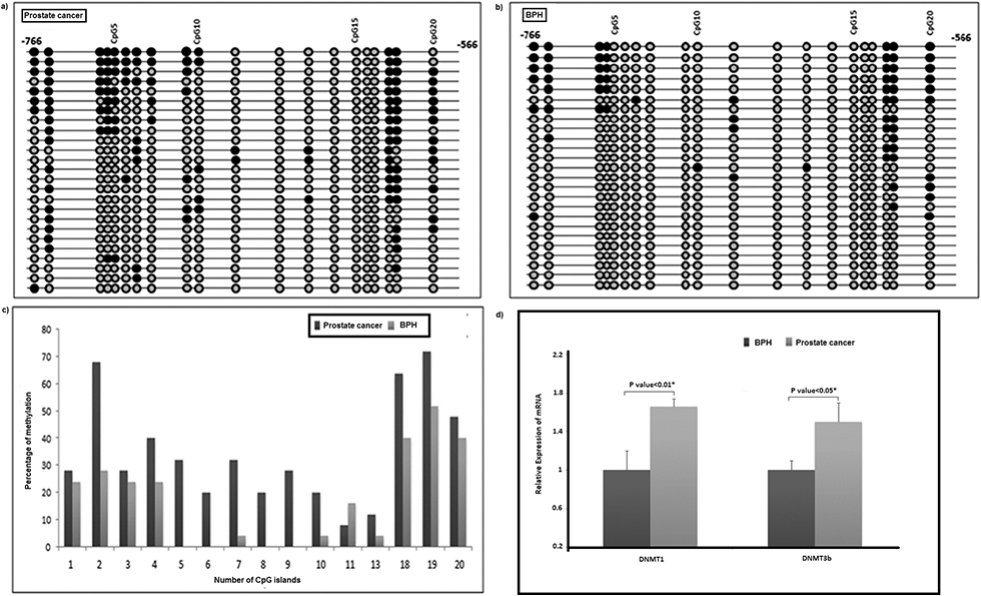
Bisulfite sequencing of *hMLH1* promoter at tissue level. (a) and (b) A ball and stick model showing differential methylation of 20 CpG sites in *hMLH1* promoter region spanning 200 bp (-766 to -566) under study in prostate cancer and BPH tissues. The grey balls represent unmethylated CpG and black balls represent methylated CpG in the string of DNA sequence. (c) Bar diagram comparing the proportion of methylation of 15 differentially modified CpGs in 50 tissues (prostate cancer: n = 25) and (BPH: n = 25). (d) Bar diagram showing elevated expression of *DNMT1* and *DNMT3b* genes in prostate cancer compared to BPH patients at mRNA level. * indicates P <0.05.

To capture the methylation pattern of CpG dinucleotides located internally to primer sequences in *hMLH1* promoter spanning -766 and -566, a region harboring 20 CpGs, was sequenced using bisulfite-modified genomic DNA from tissue samples of 50 patients (n = 25 for each group). Five CpG sites (12^th^, 14^th^-17^th^) remained unmethylated in all 50 samples. A closer look at the data revealed few distinct features in the methylation pattern distinguishing the study groups. Of 15 CpGs methylated in cancer, four (5^th^ in n = 8, 6^th^ in n = 5, 8^th^ in n = 5 and 9^th^ in n = 7) were methylated exclusively in tumor samples. The numbers of methylated CpGs in cancer and BPH samples were 15 and 11 respectively. The highest and lowest numbers of methylated CpGs in cancer samples were 12 and 1, while those in BPH patients were 7 and 0 respectively (Fig 3A–3B). The proportion of methylation was higher at ten CpG positions (1^st^-4^th^, 7^th^, 10^th^, 13^th^ and 18-20^th^) in prostate cancer samples while cytosine at 11^th^ CpG were methylated in higher proportion among BPH patients (Fig 3C). An inspection of the data revealed a critical zone spanning CpG 5^th^ to 10^th^ (-736 to -709), which was hypermethylated in prostate cancer samples (19/25 prostate cancer and 2/25 BPH tissues). Bisulfite sequencing of DNA derived from blood leukocytes from a subset of the same patient pool (15 samples from each group) revealed that CpGs in the critical zone were unmethylated in 29/30 samples. CpG at position 5^th^ of this critical zone was methylated in only one cancer samples (S3 Fig).

**Fig 4:**
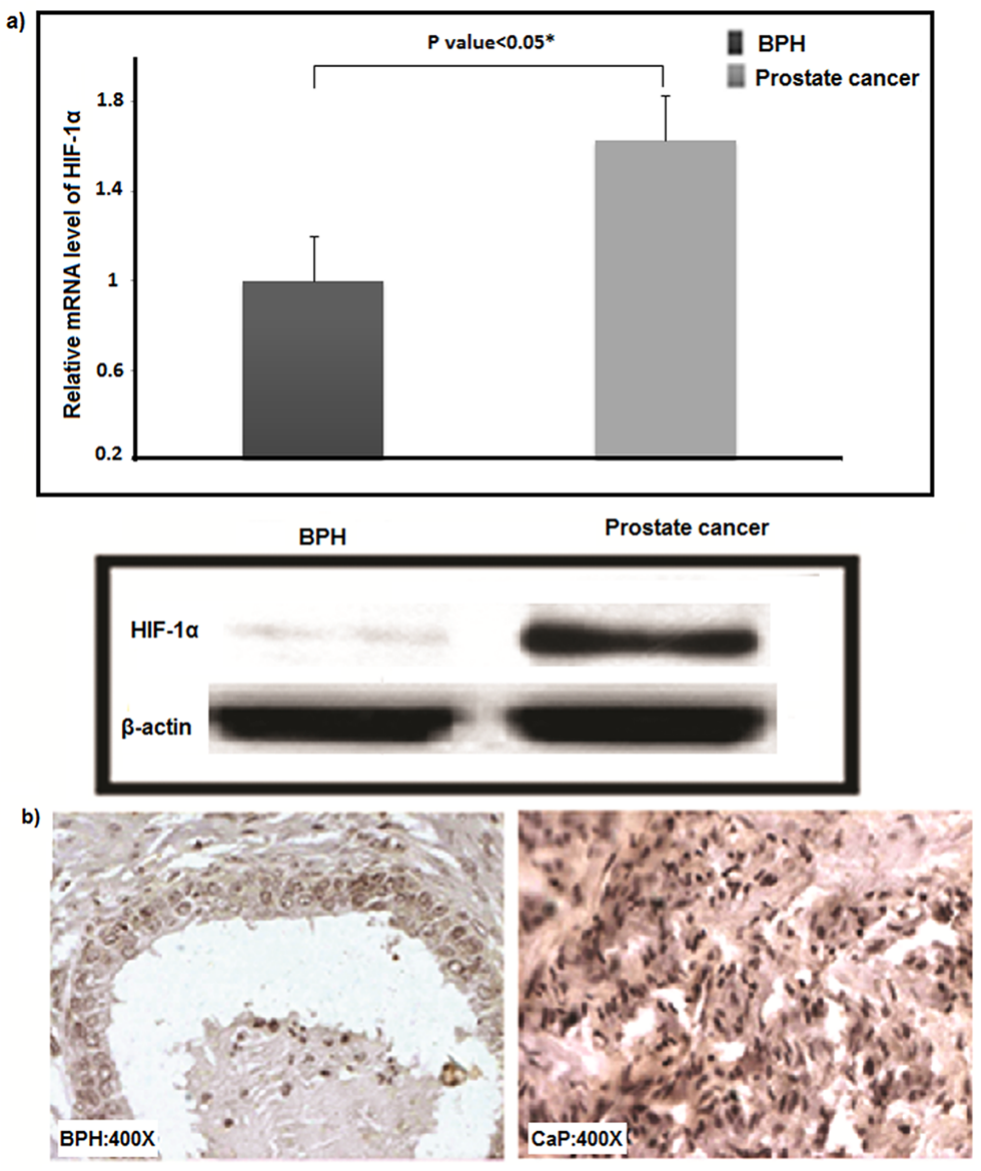
Expression of HIF-1α in malignant and benign prostatic tissues. (a) Upper panel: Bar diagram showing elevated expression of *HIF-1α* in prostate cancer compared to BPH patients at mRNA level. * indicates P <0.05. Lower panel: Representative result of Western blotting for *HIF-1α* in cancer and benign tissue of prostate where *ACTB* acted as endogenous control. **(**b) Photomicrograph of representative prostate archival specimens immune-stained with antibodies against *HIF-1α* at 400x magnification showing elevated expression of *HIF-1α* in prostate cancer tissues.

In accordance with the significantly high methylation density in prostate cancer tissues as shown by Wilcoxon Signed-rank test (P value = 0.008, Z = -2.649), a statistically significant overexpression of DNA methyl-transferase-1 (*DNMT1*) (34%, P value<0.01), de-novo-methyl-transferase (*DNMT3b*) (50%; P value<0.05) and *HIF-1α* (44%; P value<0.05) genes were observed in cancer tissues w.r.t. 18S rRNA and *ACTB* controls (Figs 3D, 4A–4B and S1). Our study revealed tissue-specific hypermethylation of *hMLH1*promoter in cancer samples.

#### MicroRNA mediated control of MMR gene expression

**Fig 5:**
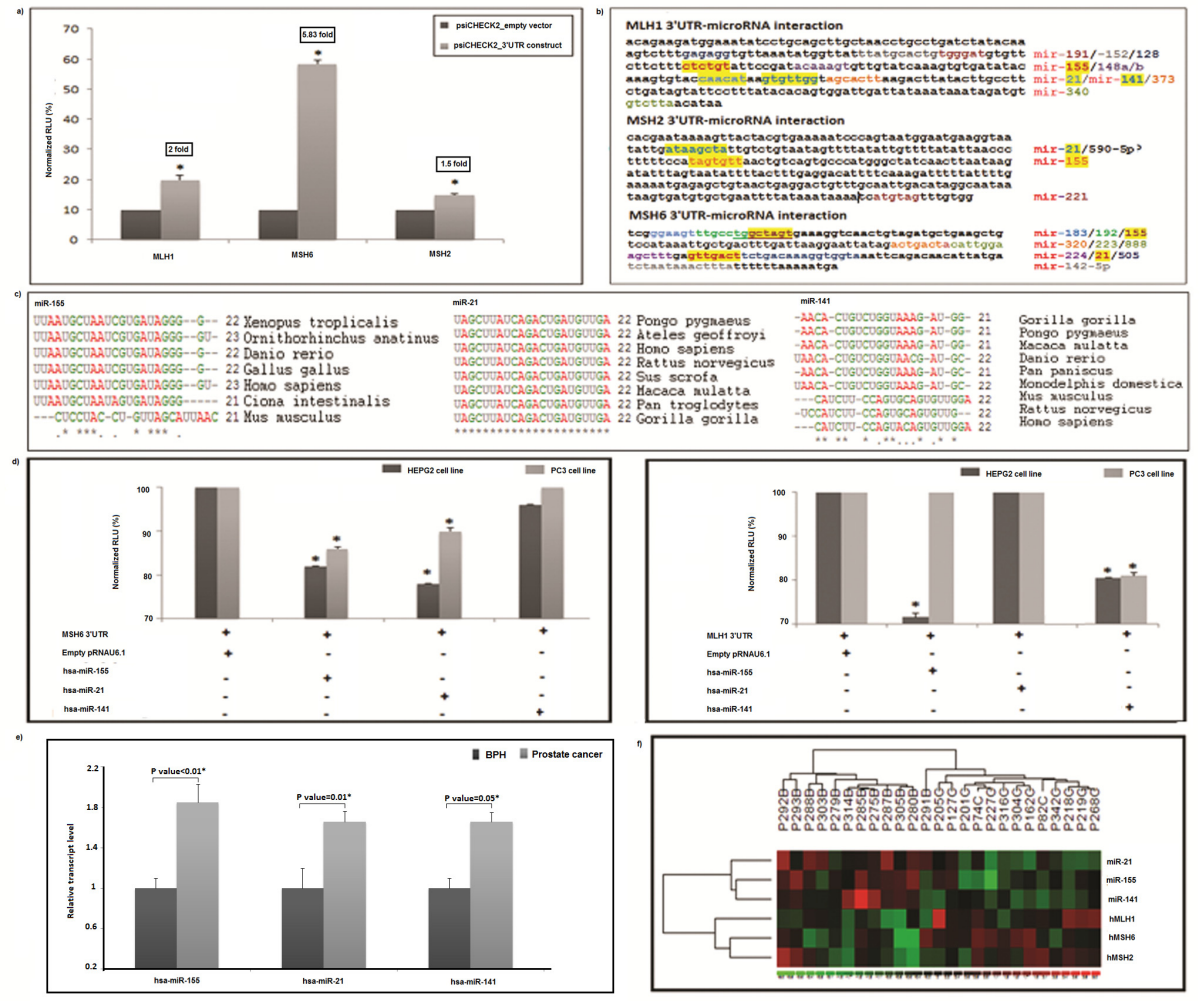
Interaction of MMR gene 3'UTRs with microRNAs. (a) Bar diagram illustrating positive 3'UTR activity of *hMLH1*, *hMSH6* and *hMSH2* genes in HEPG2 cells following reporter gene assay. Fold changes were appended in the diagram. (*) indicates statistical significance of functionality of 3'UTR regions measured in terms of P value by t-test. (b) Entire 3'UTR region of *hMLH1*, *hMSH2* and *hMSH6* were mapped for putative microRNA binding sites. The highlighted and boldfaced segments within the 3'UTR sequences represent the seed positions for the microRNAs. (c) Multiple alignments indicated that miR-155, miR-21 and miR-141 seed sequences are evolutionarily conserved across mammalian and non-mammalian species. (d) Normalized relative light units (RLU) in HEPG2 and PC3 cells were measured for *hMLH1* and *hMSH6* 3'-UTR constructs in pSiCHECK2 with (+) and without (-) the effect of microRNAs. Co-transfection of the pRNAU6.1 empty vector (+) with the 3'UTR-pSiCHECK2 constructs of *hMLH1* and *hMSH6* were set as 100% and percentage reduction in luciferase activity mediated by the three microRNAs were measured in relation to this was shown in the bar diagram. Statistical significance in terms of P values was measured with t-test. (*) indicates the statistical significance P<0.05. (e) Bar diagram showing upregulation of hsa-miR-21, hsa-miR-155 and hsa-miR-141transcripts in prostate cancer tissues compared to that of BPH patients. * indicates P<0.05. (f) Heat map showing the combination of two dendrograms displayed above and to the right. The rows represent genes on the right of the figure. Individual patient sample is shown as columns. Color represents expression level of the gene. Red represents low expression, while green represents high expression. The expression levels are continuously mapped on the color scale provided at the bottom of the figure. The dendrograms show stratification of samples into two groups: BPH (B) and prostate cancer (P), based on gene expression.

Role of microRNAs in modulating 3’UTR activity of MMR genes was also explored. The 3'UTR activity of three pSiCHECK2-constructs containing the sequences from *hMLH1* (+2305 to +2631), *hMSH6* (+3925 to +4309) and *hMSH2* (+2723 to +3108) genes were compared with that of empty pSiCHECK2 vector using DLR assay. Significantly higher reporter gene activity was detected in all three 3’UTR-constructs: *hMSH6* (5.83 fold, P value = 0.001), *hMLH1* (2 fold, P value = 0.01) and *hMSH2* (1.5 fold, P value = 0.05) (Fig 5A). A consensus microRNA map of the 3'UTRs of three genes using at least three different databases was prepared (Fig 5B). Three candidate microRNAs were selected for cell-based reporter gene assay on the basis of biological plausibility and high degree of sequence conservation in mammalian and non-mammalian vertebrate genomes (Fig 5C). To verify the binding of target microRNAs with the putative seed sequences in the 3'UTRs of MMR genes, each pre-miR construct was co-transfected in HepG2 and PC3 cells with respective pSiCHECK2-3'UTR chimera. A significant reduction in Renilla luciferase activity of pSiCHECK2-*hMSH6* by hsa-miR-155 (HEPG2: P value = 0.019; PC3: P value = 0.025) and hsa-miR-21 (HEPG2: P value = 0.0013; PC3: P value = 0.0034) constructs was detected in both cell lines. Similar reduction in Renilla luciferase activity was observed for pSiCHECK2-*hMLH1* construct by hsa-miR-141 (HEPG2: P value = 0.0013; PC3: P value = 0.02) and hsa-miR-155 (HEPG2: P value = 0.0001; PC3: P value = 0.05) constructs. The normalized RLU obtained remain unaltered when *hMSH6* and *hMLH1* 3’UTR constructs were cotransfected with hsa-miR-141 and hsa-miR-21 respectively (Fig 5D). Moreover, none of the three microRNAs under study modulated Renilla luciferase activity of pSiCHECK2-*MSH2* 3’UTR in similar transfection assays. The relative levels of hsa-miR-155 (85%; P value<0.01), hsa-miR-141 (66%; P value<0.05), and hsa-miR-21 (66%; P value = 0.01) were significantly increased in cancer tissues compared to BPH samples (Fig 5E). A heat map displaying variations of relative transcript levels of three MMR and three microRNAs genes on a color scale showed that relative expression of MMR and the microRNAs genes under study were inversely related. Above analysis stratified the study subjects in definite disease categories with exception to only one BPH sample which congregated with prostate cancer samples (Fig 5F). Pairwise comparison of transcript levels representing MMR and microRNA genes revealed an inverse correlation between *hMSH6* with hsa-miR-155 (r = -0.564; P value = 0.036) & hsa-miR-21 (r = -0.527; P value = 0.044) and between *hMLH1* with that of hsa-miR-155 (r = -0.602; P value = 0.023) and hsa-miR-141 (r = -0.531; P value = 0.049) only in cancer tissues (S4 Fig). Our study showed that upregulated oncomiRs, hsa-miR-155, hsa-miR-141 and hsa-miR-21, physically interacted with the 3’UTRs of *hMLH1* and *hMHS6* genes to disrupt their expressions.

## Discussion

Genome-wide and candidate gene based association studies have identified an unprecedented number of common variants influencing disease risk in complex disorders. Despite this accomplishment, the variants identified by these studies have, in general, explained only a small fraction of the heritable component of disease risk, and have not pinpointed with precision the causal variant(s) at the associated loci. This is partly because of our inability to establish clear connection between associated genetic variant with known disease biology [38, 39]. Biological pathway-based analysis that tests whether a set of genes from a pathway is associated with a disease trait can serve as a complementary approach [40]. Furthermore, gene expression, genotyping and other types of molecular data can be leveraged to characterize the dysregulated pathway in a disease process to accelerate the progress from genetic studies to biological knowledge that can steer the development of predictive, preventive, or therapeutic measures. Keeping in line with this, the present study summarizes our effort to detect prostate cancer related molecular events/factors that are amenable to specific management strategies.

Our population-based study recorded a concerted loss of expression of *hMLH1*, *hMSH6* and *hMSH2* genes in majority of the prostate cancer samples examined. The possible genetic and epigenetic factors underlying the observed MMR gene deficiency in prostate cancer was subsequently investigated. Our findings suggested that the downregulation of *hMLH1* gene was associated with promoter hypermethylation of a critical zone encompassing 6 CpG dinucleotides and overexpression of microRNAs such as hsa-mir-155 and hsa-mir-141 interacting with its 3’ UTR. It also showed that disruption of *hMSH6* gene expression was mediated through microRNAs, hsa-mir-155 and hsa-mir-21. In addition, an association of *hMSH6* Pro92Pro with prostate cancer was also registered. However, the effect size of this SNP in our population was weak and any biological role of this variant could not be clearly ascertained in the present study setup.

Deregulation of transcriptional machinery by hypermethylation of promoter CpGs and microRNAs are observed in a wide variety of cancers [41–46]. Our study recorded an inverse correlation between *hMLH1* transcript level and its promoter methylation quotient in the prostate cancer patients together with a distinctly higher methylation density. Similar findings have also been reported for HNPCC and gastric cancers [47, 48]. To delineate the molecular events in prostate cancer, we used BPH as an age-matched non-malignant control. BPH and prostate cancer often coexist and are major sources of morbidity in older men. Approximately 90% of men between 50–80 years of age have LUTS and a significant proportion of these patients suffer from hyperplasia of prostate gland [49, 50]. Identification of molecular criteria distinguishing the diseases early will be beneficial given the improved life expectancy in the modern scenario. Prostate cancer is a multi-focal disorder displaying histological and molecular heterogeneities which may arise due to presence of unique combination of somatic changes, genetic and/or epigenetic, some of which drive tumor development [51]. A diverse range of epigenetic heterogeneities characteristic of prostate cancer and BPH have been reported in several studies. For example, GSTP1 silencing was the most frequently detected epigenetic alterations characteristic of over 90% prostate cancer samples but rarely detected in the BPH/normal tissues [52]. Again, promoters of ER1 and ER2 were differentially methylated in prostate cancer and BPH samples with the extent of methylation being significantly higher in cancer tissues [53, 54]. On the other hand, calcium binding protein S100A2 functioning as tumor metastasis protein has been reported to be methylated in 75% cancer tissues and in 100% BPH tissues [55]. Furthermore, frequency and extent of aberrant methylation appears to increase with age in normal cell population. Consistent age-related methylation changes have been observed in normal prostate and breast tissues and in colonic mucosa [56–58]. These confounding factors that weaken the molecular signals would be ideally resolved by use of age-matched disease-free prostate tissues as control. As an alternative to normal tissues, in this study, we have analyzed methylation pattern of *hMLH1* promoter from DNA isolated from blood leukocytes in a panel of 15 BPH patients and 15 cancer patients without metastasis. This enables us to identify a central zone spanning (-736 to -709) typically hypermethylated in prostate cancer. The CpGs located on the either side of this critical zone were consistently methylated irrespective of disease type and tissue of origin and hence appear to result from age-related changes. To investigate probable functional significance of this differentially methylated zone, the sequence (28 bp) was subjected to BLAT analysis using the transcription factor ChIP data available on ENCODE (http://genome.ucsc.edu/ENCODE/). Of the several transcription factors identified, a few namely E2F1, MYC, YY1, CHD1 and SMARCA4 which have been shown to express in prostate cancer tissues are of particular interest. Since ChIP data from ENCODE does not include any cell lines from prostate epithelial origin, future studies examining the efficacy of binding of any of the above transcription factors with this DMR are needed to decipher its causal role in *hMLH1* downregulation.

In our study, the heat map analysis based on relative levels of MMR and microRNA gene expression, clustered prostate cancer and BPH patients as two independent disease entities indicating the a specific association of these microRNAs with prostate cancer. Although our study concentrated on patients’ biopsy specimens, serum levels of hsa-miR-155, hsa-miR-141 and hsa-miR-21 were reported to correlate the clinical stages in different cancers such as lung adenocarcinoma, breast cancer and NSCLC with moderate sensitivity and specificity [59–63]. To this end, our findings pave the ground for prospective studies to fine-tune the applicability of this microRNA panel as one of the components of risk prediction tool.

Tumor microenvironments, characterized by low pH, nutrient deprivation, and hypoxia have an unequivocal role in shaping malignant progression [64]. The molecular mechanisms of response to hypoxia are extremely complex, a key role being played by a transcriptional regulator, hypoxia-inducible factor (HIF), which orchestrates the expression of a wide variety of epigenetic factors like DNA methyltransferases and oncomiRs such as hsa-miR-155, hsa-miR-21, hsa-miR-424, hsa-miR-210 and hsa-miR-373[65–69]. Putative consensus sequence for HIF1- α binding site, termed as hypoxia responsive element (HRE) has been identified in the promoter region of each of hsa-miR-155 (-7521bp, -8018bp), DNMT1 (-619bp) and DNMT3b (-2467bp, -4505bp, -4509bp) (http://alggen.lsi.upc.es/). And, HIF1- α has been demonstrated to interact with the target promoter region through the HRE element modulating their gene expressions in intestinal epithelial and colorectal cells as well as in cardiac fibroblasts [67, 70, 71]. Upregulated HIF1-α in prostate cancer tissues as observed in the present study could likely to be responsible for induction of promoter hypermethylation and hsa-miR-155 mediated modulation of 3’UTR activity of *hMLH1* and *hMSH6* genes as detected by others [72, 73].

Epidemiological screening of seven SNPs located in the promoter and coding region of MMR genes has been conducted to explore any possible role of genetic variants on the observed MMR genes downregulation in prostate cancer. These SNPS have been previously implicated with susceptibility of different cancers and other phenotypes in Indian and non-Indian populations. *hMLH1* -93G>A polymorphism (rs1800734) was identified to confer significant risk for prostate cancer in North Indian population (n = 331) [74]. *hMLH1* SNP rs1799977 was found to be associated with aggressive form of prostate cancer in a study population of 1,458 Caucasian and African-American men from King County, Washington [75]. The *hMSH2* -118T>C polymorphism (rs2303425) was significantly associated with an increased risk of gall bladder in North Indian population [76]. The *hMSH6* promoter SNP rs3136228 (-557G>T) was reported to be significantly associated with increased risk of neutropenia in a panel of 154 colorectal cancer patients from Italy [77]. *hMSH6* SNP rs1042821 showed a significant trend (P_trend_<0.001) associating younger age at diagnosis (<50) in breast cancer cases in North Carolina [78]. *hMSH6* Pro92Pro polymorphism (rs1800932) showed a protective effect for tumors in the colorectum and colon in patients with Swedish origin (n = 1103 for colon cancer, n = 637 for rectal cancer, and n = 436 for controls) with the rare allele being associated with increased levels of mRNA and ovarian aging [79, 80]. In this study, the genotypes carrying the variant allele of Pro92Pro polymorphism was associated with reduced risk to prostate adenocarcinoma. Pro92Pro polymorphism has been reported to be an expression-quantitative-trait-locus for *hMSH6* in colorectal cancer [81]. To search for an extended haplotype that may harbor a putative cis-regulatory SNP, a linkage disequilibrium map around this locus was constructed using the data from GIH population (Gujarati Indians from Houston, TX) of 1000 genomes project (http://browser.1000genomes.org/Homo_sapiens). Pro92Pro was found to be in strong linkage disequilibrium with its downstream variants such as rs1800935 (r^2^ = 0.89) and rs2020911 (r^2^ = 0.99) but not with any of the upstream variants including rs3136228 and rs1042821 we studied. Pairwise r^2^ estimates of these two comparisons in our population were 0.34 and 0.41 respectively. However, any direct genetic impact of this low effect size polymorphism could not be drawn due to small sample size in the present replication cohort. Further studies with a larger sample-size and a comprehensive annotation of mutational landscape of *hMSH6* gene are required to connect the observed genetic protection imposed by *hMSH6* gene with prostate cancer [82].

Collectively, this population-based study establishes that the deficiency of *hMLH1* and *hMSH6* genes is one hallmark differentiating prostate cancer from BPH. It also presents statistical and molecular evidences that disruption of above genes is correlated with promoter hypermethylation and upregulation of oncomiRs, hsa-miR-155, in particular. To the best of our knowledge, this study presents the most comprehensive research on MMR deficiency in prostate cancer patients in India. In absence of a robust population based surveillance program, majority of the carcinogenesis cases gets detected either at the advanced stage or during treatment for BPH. Since BPH and prostate cancer are both age-related cyto-proliferative diseases with the similar hormones and inflammatory processes playing crucial roles in their development, our attempt to determine a differential molecular attribute which could be used as a predictive sign of prostate cancer has a significant clinical relevance.

## Supporting Information

S1 Table Sequences and product sizes of the primers used in Real Time PCR and cloning experiments (DOC)

S2 Table Primer sequences, PCR conditions, and restriction endonucleases used in genotyping of single nucleotide polymorphisms. (DOC)

S1 Fig MMR gene expression w.r.t. *β-act*in in BPH and prostate cancer tissues. Bar diagram showing relative expression at transcript level of (a) *hMLH1*, *hMSH6* and *hMSH2*, (b) *DNMT1* and *DNMT3b* and (c) *HIF1-α* in prostate cancer tissues compared to BPH with *ACTB* as endogenous control. * indicates P<0.05
(TIF)

S2 Fig Methylation specific PCR for *hMLH1*and *hMSH2* with controls. Primer sets for amplification were designated as unmethylated (U) and methylated (M). Corresponding lanes are: 100 bp plus ladder (L) in lane number 1, 11. Water was used as negative control for each PCR reaction (lanes 2, 3). SssI methyl transferase treated normal lymphocytes (NL) was used as positive control for methylation (lanes 4, 5). Bisulfite modified DNA from representative BPH (lanes 6, 7) and CaP (lanes 8, 9) tissues were amplified in MSP. Unmodified DNA from normal lymphocyte serves as negative control for methylation (lane 10).
(TIF)

S3 Fig Bisulfite sequencing of *hMLH1* promoter at tissue level. (a) and (b) A ball and stick model showing differential methylation of 20 CpG sites in *hMLH1* promoter region spanning 200 bp (-766 to -566) under study in blood lymphocytes from 15 prostate cancer and 15 BPH patients. The grey balls represent unmethylated CpG and black balls represent methylated CpG in the string of DNA sequence. Portion of the sequence in blue box indicates the 6 CpG dinucleotides that cover a critical zone typically methylated in prostate cancer.
(TIF)

S4 Fig Correlation between MMR genes and microRNAs. Upper panel: Plots showing correlation between transcript level of *hMSH6* with that of hsa-miR-155 and hsa-miR-21 in prostate cancer. Lower panel: Plots showing correlation between transcript level of *hMLH1*with that of hsa-miR-155 and hsa-miR-141 in BPH. Pearson’s correlation coefficient (r) and P values are indicated for each test. * indicates P<0.05.
(TIF)
