# Supplementary material for: A Study of Molecular Signals Deregulating Mismatch Repair Genes in Prostate Cancer Compared to Benign Prostatic Hyperplasia
Source: PLoS One. 2015 May 4;10(5):e0125560. doi: 10.1371/journal.pone.0125560 (PMC4418837; doi:10.1371/journal.pone.0125560)
Supplement: S1 Table — (DOC) [file pone.0125560.s001.doc]

**Supplementary Materials**

**Table S1: Sequence and product size of the primers used in Real Time PCR and cloning experiments**

| Oligo Name | Oligonucleotide (5'-3') sequences | Product size |
| --- | --- | --- |
| 18s rRNA | FP:GTAACCCGTTGAACCCCATT  RP:CCATCCAATCGGTAGTAGCG | 151bp |
| β-actin | FP:GCGGGAAATCGTGCGTGACATT  RP:GATGGAGTTGAAGGTAGTTTCGTG | 232bp |
| MLH1 | FP:TGCTGGCAATCAAGGGACCCAGAT  RP:CACGGTTGAGGCATTGGGTAGTGT | 214bp |
| MSH2 | FP:GGAGCACCTGTTCCATATGTACGA  RP: TTGGGGCCAGTAATGATGTG | 175bp |
| MSH6 | FP:AAATGCTGAAGAACGGAGGGATG  RP:GACTATACTTAGCCCAGGCACAGT | 146bp |
| DNMT1 | FP:TCTTCGGCAACATCCTGGACAAG  RP:ATCAGTGCATGTTGGGGATTCCTG | 198bp |
| DNMT3b | FP:AGCTGCAGGACTGCTTGGAATACA  RP:CATGTTGGACACGTCTGTGTAGTG | 113bp |
| HIF1-α | FP:CTAACGTGTTATCTGTCGCTTTG  RP:CCTACTGCTTGAAAAAGTGAACC | 136bp |
| MLH1-Ext-FP | GAGTAGTTTTTTTTTTAGGAGTGAAG | 199bp |
| MLH1-Ext-RP | AAAAACTATAAAACCCTA TACCTAATCTA |
| MLH1-Int-UF | TTTTGATGTAGATGTTTTATTAGGGTTGT | 114bp |
| MLH1-Int-UR | ACCACCTCATCATAA CTACCCACA |
| MLH1-Int-MF | ACGTAGACGTTTTATTAGGGTCGC | 110bp |
| MLH1-Int-MR | CCTCATCGTAACTACCCGCG |
| MSH2-UF | AGTTAAAGTTATTAGTGTGTGTGG | 202bp |
| MSH2-UR | TCCTAATTAAAAAAAATACACAAC |
| MSH2-MF | AGTTAAAGTTATTAGCGTGCG | 200bp |
| MSH2-MR | CTAATTAAAAAAAATACGCGAC |
| MSH6-UF | TTTGTGTGGGGTTTAGGTGTT | 368bp |
| MSH6-UR | AACCTTATTAACATCACTCAA |
| MSH6-MF | TTCGCGCGGGGTTTAGGCGTC | 368bp |
| MSH6-MR | AACCTTATTAACATCACTCAA |
| MLH1-FP-Xho1 | TGACTCGAGGGAAGGGAACCTGATTGGA | 326bp |
| MLH1-RP-Not1 | CAAGCGGCCGCTCCACTGTGTATAAAGGAATAC |
| MSH2-FP-Xho1 | CAGCTCGAGAAAGCCCTGGAACTTGA | 485bp |
| MSH2-RP-Not1 | TCAGCGGCCGCATTGCAAACAGTCCTCAG |
| MSH6-FP-Xho1 | AAACTCGAGTGTTGCTGTGCGCCTA | 352bp |
| MSH6-RP-Not1 | CCAGCGGCCGCCCTTTGTCAGAAGTCAACTC |
| miR21-FP-BamH1 | GGAGGATCCGCATTATGAGCATTATGTCAG | 351bp |
| miR21-RP-HindIII | TAAAAGCTTGTGCCACCAGACAGAAGGAC |
| miR155-FP-BamH1 | TGCGGATCCTGTCACTCCAGCTTTATAAC | 364bp |
| miR155-RP-HindIII | GGTAAGCTTTGAACATCCCAGTGACCAGA |
| miR141-FP-BamH1 | AGTGGATCCGCGATTTGTCACCTGGTGGA | 385bp |
| miR141-RP-HindIII | CTGAAGCTTCAGCTGTAAAGGCAGCCAT |

FP: Forward primer, RP: Reverse primer, Ext: External, Int: Internal, MF & MR: Methylated forward and methylated reverse primer respectively, UF & UR: Unmethylated forward and Unmethylated reverse primer respectively, bp: base pairs.
