# Supplementary material for: A Study of Molecular Signals Deregulating Mismatch Repair Genes in Prostate Cancer Compared to Benign Prostatic Hyperplasia
Source: PLoS One. 2015 May 4;10(5):e0125560. doi: 10.1371/journal.pone.0125560 (PMC4418837; doi:10.1371/journal.pone.0125560)
Supplement: S2 Table — (DOC) [file pone.0125560.s002.doc]

**Supplementary Materials**

**Table S2: Primer sequences, PCR conditions, and restriction endonucleases used in genotyping of single nucleotide polymorphisms.**

| **db SNP**  **rs ID** | **Restriction enzymes**  **/sequencing** | **Annealing temperature** | **Oligonucleotide (5'-3') sequences** | **Product**  **size** |
| --- | --- | --- | --- | --- |
| **MLH1** **rs1800734** | PvuII | 60°C | FP:GCAAGCGCATATCCTTCTAGGTAG  RP:CCGTTAAGTCGTAGCCCTTAAGTG | 413bp |
| **MLH1 rs1799977** | Sequencing | 60°C | FP:TCCAGGTATTCAGTACACAATG  RP:ATGTGATGGAATGATAAACCAAG | 351bp |
| **MSH2**  **rs2303425** | Bsr1 | 58°C | FP:GCTTCAAGCCTTGCAGCTGAGT  RP:CTCCTCACCTCCTGGTTGAAGA | 306bp |
| **MSH2**  **rs6753135** | TspRI | 58°C | FP:CTATTGCACTGACAGAGGGAGTG  RP:CTCCTAGGTTCCAAGGTGTCCTG | 285bp |
| **MSH6**  **rs3136228** | MspI | 65°C | FP:GGCTCAGATAACGGACTGTGG  RP:ACCCGAAAGGCCTCGGAAAG | 355bp |
| **MSH6**  **rs1042821** | NciI | 58°C | FP:TTAGGAGCTCCGTCCGACAGAAC  RP:CCTCCGTTGAGGTTCTTCGCCTT | 270bp |
| **MSH6**  **rs1800932** | NciI | 60°C | FP:CCTGCCATCAGCATTATACCA  RP:CTGTACATGAACACGGACTGA | 434bp |

| FP: Forward primer, RP: Reverse primer, bp: base pair. |  |  |
| --- | --- | --- |
