# Supplementary figures and images for: A Study of Molecular Signals Deregulating Mismatch Repair Genes in Prostate Cancer Compared to Benign Prostatic Hyperplasia
Source: PLoS One. 2015 May 4;10(5):e0125560. doi: 10.1371/journal.pone.0125560 (PMC4418837; doi:10.1371/journal.pone.0125560)

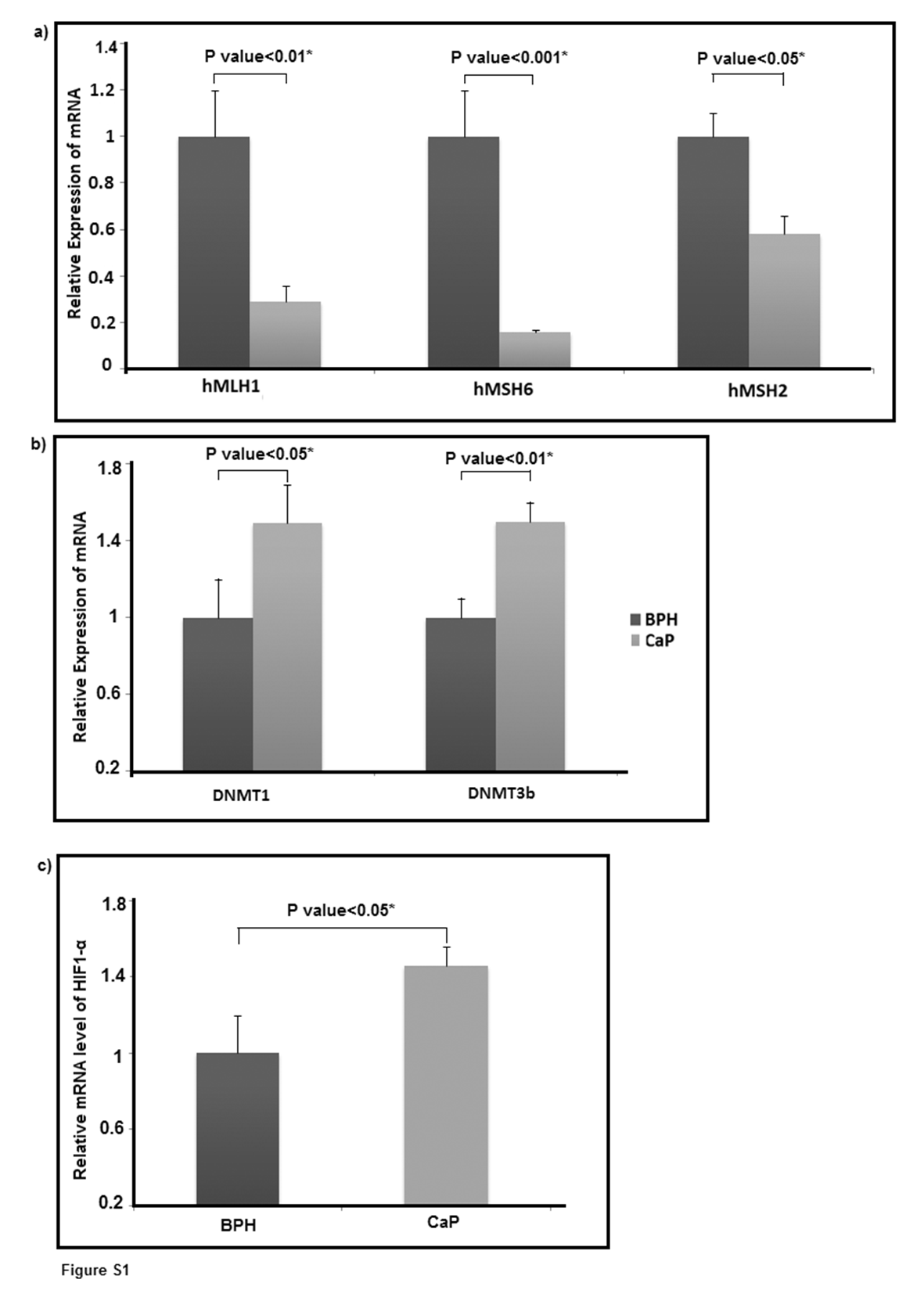

Supplement: S1 Fig — Bar diagram showing relative expression at transcript level of (a) hMLH1, hMSH6 and hMSH2, (b) DNMT1 and DNMT3b and (c) HIF1-α in prostate cancer tissues compared to BPH with ACTB as endogenous control. * indicates P<0.05 (TIF) [file pone.0125560.s003.tif]

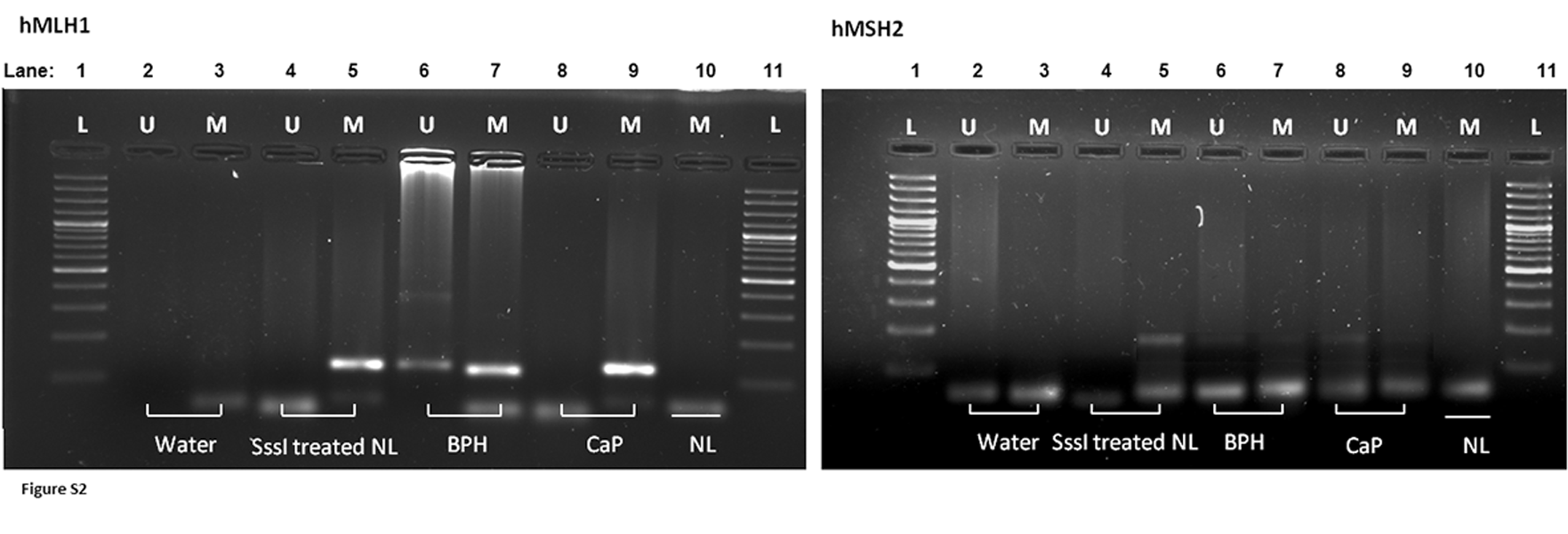

Supplement: S2 Fig — Primer sets for amplification were designated as unmethylated (U) and methylated (M). Corresponding lanes are: 100 bp plus ladder (L) in lane number 1, 11. Water was used as negative control for each PCR reaction (lanes 2, 3). SssI methyl transferase treated normal lymphocytes (NL) was used as positive control for methylation (lanes 4, 5). Bisulfite modified DNA from representative BPH (lanes 6, 7) and CaP (lanes 8, 9) tissues were amplified in MSP. Unmodified DNA from normal lymphocyte serves as negative control for methylation (lane 10). (TIF) [file pone.0125560.s004.tif]

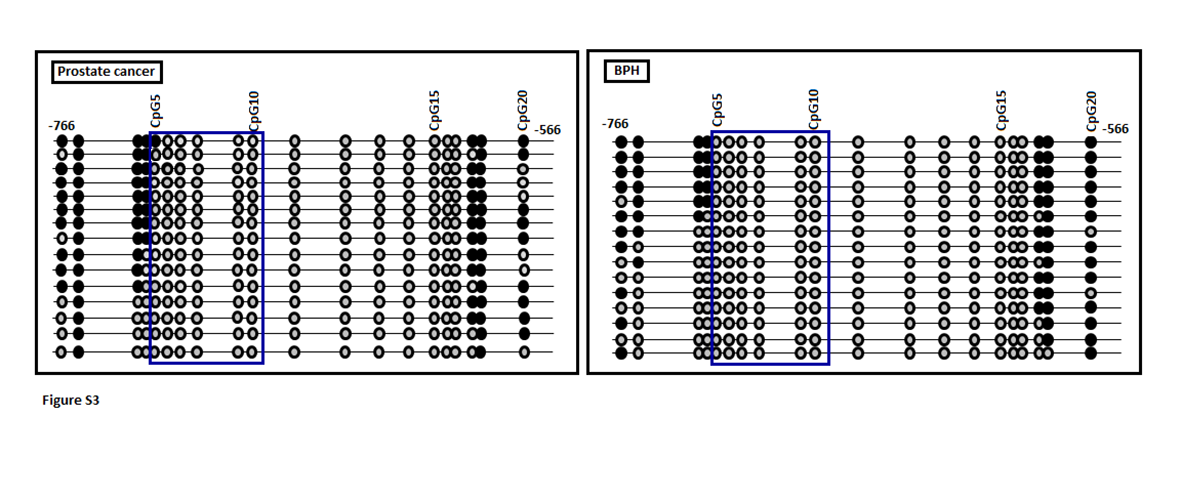

Supplement: S3 Fig — (a) and (b) A ball and stick model showing differential methylation of 20 CpG sites in hMLH1 promoter region spanning 200 bp (-766 to -566) under study in blood lymphocytes from 15 prostate cancer and 15 BPH patients. The grey balls represent unmethylated CpG and black balls represent methylated CpG in the string of DNA sequence. Portion of the sequence in blue box indicates the 6 CpG dinucleotides that cover a critical zone typically methylated in prostate cancer. (TIF) [file pone.0125560.s005.tif]

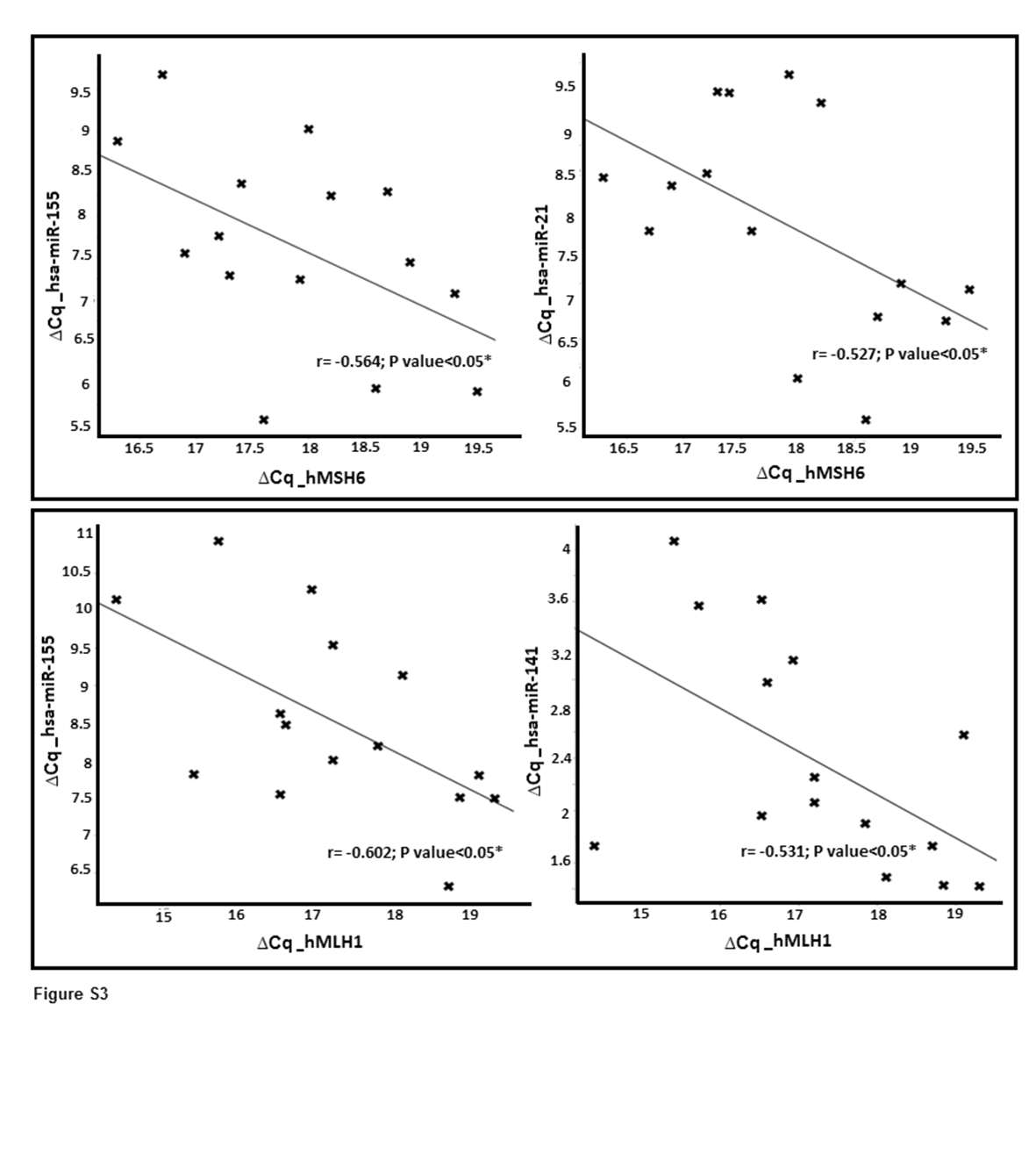

Supplement: S4 Fig — Upper panel: Plots showing correlation between transcript level of hMSH6 with that of hsa-miR-155 and hsa-miR-21 in prostate cancer. Lower panel: Plots showing correlation between transcript level of hMLH1with that of hsa-miR-155 and hsa-miR-141 in BPH. Pearson’s correlation coefficient (r) and P values are indicated for each test. * indicates P<0.05. (TIF) [file pone.0125560.s006.tif]
